# Neurotensin receptors in pancreatic ductal carcinomas

**DOI:** 10.1186/s13550-015-0094-2

**Published:** 2015-03-24

**Authors:** Meike Körner, Beatrice Waser, Oliver Strobel, Markus Büchler, Jean Claude Reubi

**Affiliations:** Cell Biology and Experimental Cancer Research, Institute of Pathology, University of Berne, PO Box 62, Murtenstrasse 31, CH-3010 Berne, Switzerland; Department of General, Visceral and Transplantation Surgery, University Hospital Heidelberg, Heidelberg, Germany

**Keywords:** Neurotensin receptors, Pancreatic ductal carcinomas, Liver metastasis, Pancreatic intraepithelial neoplasia, Tumor imaging

## Abstract

**Background:**

The frequent expression of neurotensin receptors (NT-R) in primaries of pancreatic ductal carcinomas has triggered the development of radioactive neurotensin analogs for possible *in vivo* targeting of these tumors. However, the complete lack of information regarding NT-R in liver metastases of pancreatic cancer and pancreatic intraepithelial neoplasia (PanIN) makes an *in vitro* study of NT-R in these tissues indispensable.

**Methods:**

Using *in vitro* receptor autoradiography with ^125^I-[Tyr^3^]-neurotensin, NT-R were investigated in 18 primaries and 23 liver metastases of pancreatic ductal carcinomas as well as in 19 PanIN lesions.

**Results:**

We report here that 13 of 18 ductal carcinoma primaries and 14 of 23 liver metastases expressed NT-R. Moreover, none of the six PanIN 1B cases expressed NT-R, while two of six PanIN 2 and five of seven PanIN 3 expressed NT-R. Binding was fully displaced by the type 1 NT-R-selective antagonist SR48692, indicating that the NT-R in the tumors are of the type 1 NT-R subtype.

**Conclusions:**

These *in vitro* data extend the currently available information on NT-R in invasive and non-invasive pancreatic ductal tumors. They suggest that type 1 NT-R may be a novel, specific marker of PanIN of higher degree. The high expression of NT-R in primaries and metastases of invasive cancer strongly support the need to develop radioactive neurotensin analogs for the diagnosis and therapy of this tumor type.

## Background

Two decades ago, neurotensin receptors (NT-R) were found to be expressed in approximately 75% of primary pancreatic ductal adenocarcinomas by NT-R autoradiography [1]. The NT-R were present in high density in the tumor cells, while the non-neoplastic pancreatic ducts did not express NT-R [1]. This suggested for the first time the possibility to specifically target pancreatic tumor cells via NT-R [1]. These expression data were confirmed by Ehlers et al. showing an approximately 90% incidence of NT-R in pancreatic cancers by RT-PCR measurement [2]. Later on, several groups identified NT-R in various other cancer types [3-7]. The role and function of the NT-R in cancer has also been investigated in the past one to two decades [8,9]. It appears to be clear that NT-R (especially the type 1 NT-R (also named NTS1 [10])) mediate a proliferative action of neurotensin in pancreatic cancer and also in other tumors [9,11-14]. There is also strong evidence that the type 1 NT-R-selective antagonist SR48692 [15] can inhibit tumoral proliferation [12-14]. Therefore, the use of neurotensin analogs has been proposed as strategy for the antiproliferative treatment of pancreatic cancer [3,8,16,17].

On the other hand, these *in vitro* receptor expression data [1,2] also triggered the development of radioactive neurotensin analogs that could be used for the visualization and, eventually, targeted radiotherapy of pancreatic ductal adenocarcinoma and other tumors. The majority of the published compounds are radiolabeled NT-R agonists [18-24], but radiolabeled NT-R antagonists have recently appeared [25,26]. Proof of principle that pancreatic ductal carcinomas can be visualized in patients with radiolabeled NT-R agonists was provided in preliminary studies [27-29]. Indeed, it would be important to have a reliable non-invasive tool permitting to visualize the majority of pancreatic ductal carcinomas in patients. It would be equally important to be able to treat the patients that have a receptor-positive carcinoma by giving them a radiotherapeutic dose of the radiolabeled neurotensin; many of the patients diagnosed with pancreatic ductal carcinomas already have or will soon have metastatic disease. Most patients die due to systemic disease within a short period of time after resection [30].

A targeted therapy of pancreatic cancer would, however, make sense only if all tumor manifestations in a given patient, namely the primary and the metastases, express NT-R. Considering the frequent observation of tumor heterogeneity in cancer in general, in terms of both histopathological features and of biological parameters, it is not possible to simply extrapolate that NT-R-positive primary pancreatic ductal carcinomas will give rise to NT-R-positive metastases. Unfortunately, there are presently no experimental data indicating whether metastases of ductal pancreatic carcinomas express NT-R. This lack of information is due in part to the fact that these metastases are not operated and that metastasis material is hardly available for *in vitro* investigations. In the present study, we have been able to investigate a significant number of metastases of ductal pancreatic carcinomas, sometimes together with their primaries, using NT-R autoradiography. To complete the study, we have also investigated NT-R in non-invasive, intraductal neoplasia (PanIN) using the same methodology.

## Methods

### Tissues

Pancreatic ductal carcinoma samples were collected at the Department of General, Visceral and Transplantation Surgery, University Hospital Heidelberg, Heidelberg, Germany, and at the Institute of Pathology, University of Bern, Bern, Switzerland. The number and types of tissues investigated are listed in Tables 1 and 2. All tissues were frozen in liquid nitrogen or in dry ice immediately after surgical resection and stored at −70°C. Informed consent was available for all patients. The study collection conformed to the ethical guidelines of both centers and was reviewed by the Institutional Review Boards of University Hospital Heidelberg and University of Bern.

**Table 1:** **NT receptors in ductal pancreatic cancer primaries and liver metastases**

**Patient number**	**Primary**	**Metastasis**	**Comments**
	**Receptor density dpm/mg tissue**	**Receptor density dpm/mg tissue**	
Primary only			
1	8,335 (9,392; 7,278)^a^		
2	0		
3	0		
4	6,194 (6,317; 6,071) het^a^		
5	9,600 (9,301; 9,899)^a^		
Primary + metastasis of same patient			
6	9,310 (9,157; 9,462) het^a^	0	
7	3,053 (2,953; 3,152) het^a^	3,982 (4,584; 3,379) het^a^	
8	10,274 (10,626; 9,921)^a^	5,084 (5,174; 4,993) het^a^	
9	0	0	Chemotherapy before resection
10	6,026 (6,243; 5,808)^a^	0	
11	6,145 (6,405; 5,884)	4,482 (4,978; 3,986)	
Metastasis only			
12		10,866 (11,013; 10,719)^a^	
13		8,611 (8,189; 9,033)^a^	
14		8,517 (8,791; 8,243)^a^	
15		0	
16		0	
17		6,124 (6,075; 6,172) het^a^	Chemotherapy before resection
18		13,207 (13,169; 13,245)^a^	
19		13,235 (13,047; 13,422)^a^	
20		9,753 (9,872; 9,634)^a^	
21		0	
22		6,842 (8,250; 5,433)^a^	
23		10,684 (12,298; 9,070)^a^	
24		0	
25		0	
26		7,671 (7,259; 8,083)	
27		7,828 (7,959; 7,697)	
28		0	
Mean ± SEM of positive cancers	7,367 ± 860	8,349 ± 791	
Total incidence	8/11 (73%)	14/23 (61%)	

het, heterogeneous NT-R distribution. Values represent NT-R density (dpm per mg tissue) as mean of two separate experiments with individual values in parenthesis. ^a^In addition to the displacement by neurotensin, full displacement by SR48692 was observed in these tumors.

**Table 2:** **Quantification of NT-R in PanIN 1B, 2, and 3 and in concomitant invasive pancreatic carcinoma**

**Patient number**	**PanIN 1B**	**PanIN 2**	**PanIN 3**	**Invasive ductal carcinoma** ^**b**^
29	-	1,882 (1,732;1,923)^a^	-	-
30	-	-	5,809 (5,937;5,681)^a^	1,257 (1,290;1,223)^a^
31	0	-	-	-
32	0	0	0	0
33	-	>10,000 (>10,000;>10,000)^a^	9,871 (9,741;10,000)^a^	8,769 (8,885;8,652)^a^
34	0	-	1,937 (1,881;1,992)^a^	1,651 (1,759;1,542)^a^
35	-	0	3,103 (3,209;2,996)^a^	2,539 (2,771;2,306)^a^
36	0	0	-	-
37	0	-	9,988 (9,976;10,000)^a^	>10,000 (>10,000;>10,000)^a^
38	0	0	0	0
Mean ± SEM of positive cases	0	5,914 ± 4,086	6,142 ± 1,669	4,843 ± 1,876

Values represent neurotensin receptor density (dpm/mg tissue). ^a^In addition to the displacement by neurotensin, full displacement by SR48692 was observed in these tissues. ^b^These cases are different from those listed in Table 1. Hyphen (−) means no corresponding tissue available.

### NT-R autoradiography

Receptor autoradiography was performed on 20-μm-thick cryostat (Microm HM550, Thermo Fisher Scientific, Walldorf, Germany) sections of the tissue samples, mounted on microscopic slides and then stored at −20°C for at least 3 days to improve adhesion of the tissue to the slide. Sections were pre-incubated in 50 mM Tris–HCl pH 7.4 with 0.02% BSA for 3 times at 5 min at 20°C. Slides were then incubated in 50 mM Tris–HCl pH 7.4 containing 0.02% BSA, 1 mM o-phenantroline, 1 mM MgCl_2_ and 40,000 dpm/100 μl (corresponding to 90 pM) ^125^I-[Tyr 3]-Neurotensin (2,000 Ci/mmol; ANAWA, Wangen, Switzerland) for 1 h at room temperature. Additional sections were incubated in the presence of 100 nM non-radioactive neurotensin (Bachem, Bubendorf, Switzerland) to assess non-specific binding, as well as in the presence of 1,000 nM of the type 1 NT-R-selective analog SR48692 (Sanofi, Paris, France), for NT-R subtyping. After incubation, the slides were washed for 10 min at 4 °C in four consecutive baths containing 50 mM Tris–HCl pH 7.4 with 0.02% BSA. After a brief dip in buffer without BSA, the sections were dried under a stream of cold air and then exposed to Kodak (Rochester, NY, USA) films Biomax MR for 7 days at 4 °C. The autoradiograms were quantified using a computer-assisted image processing system (Analysis Imaging System, InterFocus, Mering, Germany). Tissue standards for iodinated compounds (Amersham, Aylesbury, UK) were used for this purpose [1,4].

## Results and discussion

**Figure 1:**
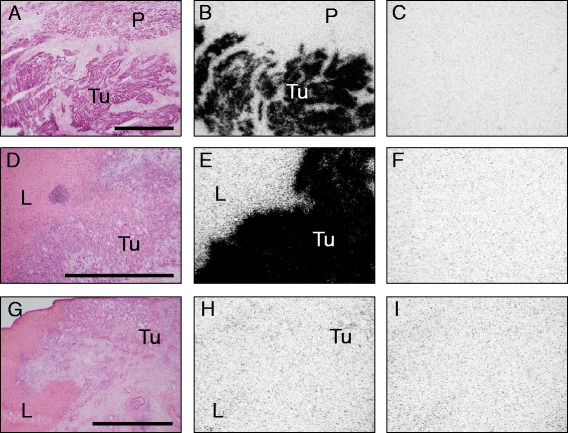
**Expression of NT-R by in vitro receptor autoradiography.** NT-R expressed in a ductal pancreatic carcinoma: primary **(A-C)** and liver metastasis **(D-F)**. As negative control, a NT-R-negative liver metastasis is also shown **(G-I). (A), (D), (G)** Hematoxylin-eosin-stained sections showing tumor tissue (Tu) and the adjacent pancreas (P) or liver (L), respectively. Bars = 1 mm. **(B)**, **(E)** Autoradiograms showing total binding of ^125^I-[Tyr^3^]-neurotensin. High density of NT-R is found in the tumors but not in the pancreas or liver. **(H)** Autoradiogram showing total binding of ^125^I-neurotensin. This metastasis lacks NT-R. **(C), (F), (I)** Autoradiograms showing non-specific binding of ^125^I-[Tyr^3^]-neurotensin (in the presence of 100 nM neurotensin).

Table 1 summarizes the data of NT-R incidence and density in pancreatic ductal carcinoma patients. The results are divided into three groups, depending on the availability of the respective frozen tissue: (1) primaries only, (2) primary and liver metastasis of the same patient, and (3) metastases only. It can be seen that not only primaries do often express NT-R in high density but also the liver metastases. The overall incidences of NT-R expression are 73% (8 of 11 cases) in primaries and 63% (14 of 23 cases) in metastases. Interestingly, all NT-R-expressing tumors have a high receptor density, namely above 3,000 dpm/mg tissue. From the six patients of whom primary and metastasis are available, three have high NT-R expression in both primary and metastasis, two have high expression in the primary only, and one is receptor-negative in both. This latter case had chemotherapy (gemcitabine) prior to the surgical resection. It cannot, however, be concluded definitely that chemotherapy is responsible for the lack of NT-R, since another chemotherapy-treated patient (Table 1) had a high NT-R expression over 6,000 dpm/mg tissue in his tumor. As also stated in Table 1, we have also performed displacement experiments with incubation of the ^125^I-[Tyr^3^]-neurotensin radioligand in the presence of 1,000 nM of the cold type 1 NT-R-selective analog SR48692. In the tested cases listed in Table 1, radioligand binding could be completely displaced by SR48692 indicating the presence of the type 1 NT-R subtype in these tissues. Three tumors (No. 11, 26, 27) could not be tested for SR48692 due to lack of tissue. Figure 1 illustrates a case of a patient with high NT-R expression in both the primary and the liver metastasis. Here, the NT-R are densely and homogeneously distributed in both tumor manifestations. The adjacent normal pancreas and liver, respectively, are NT-R-negative.

**Figure 2:**
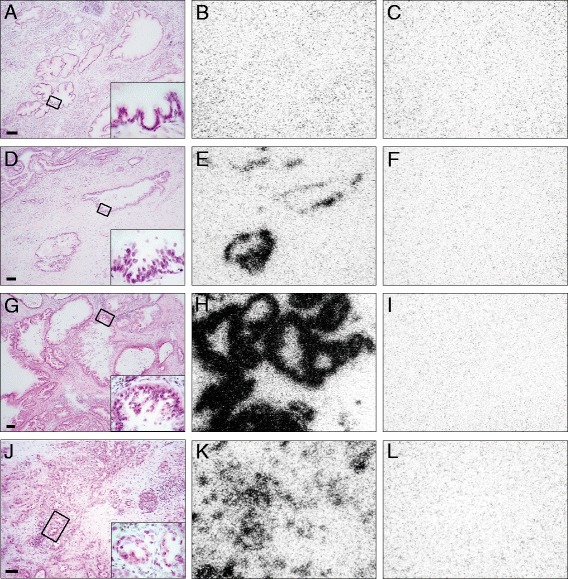
**NT-R** ***in vitro*** **receptor autoradiography in PanIN.** *In vitro* receptor autoradiography of NT-R in various PanIN, PanIN 1B **(A-C)**, PanIN 2 **(D-F)**, and PanIN 3 **(G-I)**, and in invasive cancer **(J-L)**. **(A)**, **(D)**, **(G)**, **(J)** Hematoxylin-eosin-stained sections (bars = 0.1 mm), including insert of representative area at high magnification. **(B)**, **(E)**, **(H)**, **(K)** Autoradiograms showing total binding of ^125^I-[Tyr^3^]-neurotensin. While the PanIN 1 case **(B)** is NT-R-negative, PanIN 2 **(E)**, PanIN 3 **(H)**, and invasive cancer **(K)** are NT-R-positive. **(C)**, **(F)**, **(I)**, **(L)** Autoradiograms showing non-specific binding of ^125^I-[Tyr^3^]-neurotensin (in the presence of 100 nM neurotensin).

Table 2 shows samples of additional pancreatic ductal tumor patients with resected pancreatic tissue containing various stages of PanIN with or without the corresponding invasive ductal carcinoma. Here, there is a clear difference in NT-R expression depending on the PanIN stage: None of the six PanIN 1B cases expresses NT-R, and only two out of the six PanIN 2 cases express NT-R, whereas the majority of the PanIN 3 cases (five out of seven cases) express NT-R. Interesting is the observation that in the two latter NT-R-negative PanIN 3 patients, their corresponding invasive ductal carcinomas (and their PanIN 1B and 2 stages as well) are also NT-R-negative. In three other cases, we see the NT-R expressed in PanIN 3 stages as well as in invasive ductal carcinomas, while adjacent PanIN 1B and/or PanIN 2 stages in these patients are NT-R-negative. Figure 2 illustrates the NT-R status in various PanIN lesions.

**Figure 3:**
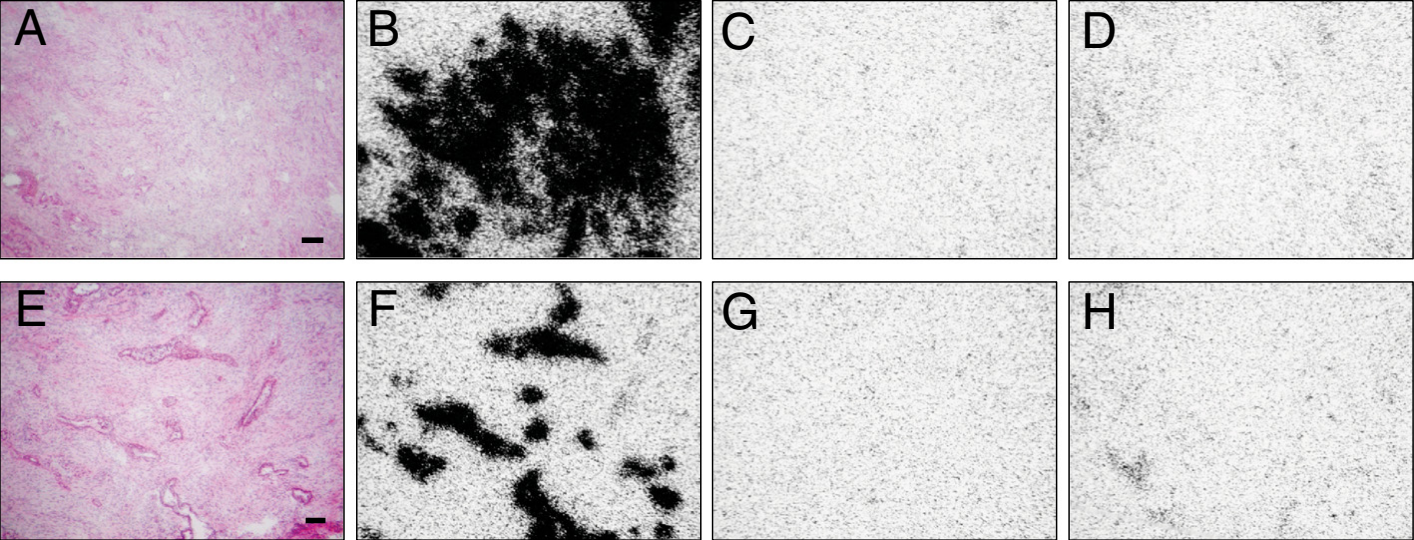
**Autoradiography of NT-R and competition by SR48692.** NT-R autoradiography of PanIN 3 **(A-D)** and of an adenocarcinoma **(E-H)** with neurotensin and the type 1 NT-R-selective SR48692 as displacer. **(A)**, **(E)** Hematoxylin-eosin-stained sections (bars = 0.1 mm). **(B)**, **(F)** Autoradiograms showing total binding of ^125^I-[Tyr^3^]-neurotensin. **(C)**, **(G)** Autoradiograms showing non-specific binding (in the presence of 100 nM neurotensin). **(D)**, **(H)** Autoradiograms showing full displacement of ^125^I-[Tyr^3^]-neurotensin binding in the presence of 1,000 nM SR48692. The detected NT-R are therefore the type 1 NT-R.

As seen in Table 2, we have performed in the NT-R-expressing PanIN 2 and PanIN 3 and invasive ductal cancer cases displacement experiments with incubation of the ^125^I-[Tyr^3^]-neurotensin radioligand in presence of 1,000 nM of the cold type 1 NT-R-selective analog SR48692. In all cases, radioligand binding could be completely displaced by SR48692, reaching low binding levels corresponding to non-specific binding, similar to that identified in the presence of 100 nM cold neurotensin. This is illustrated in Figure 3 for a PanIN 3 lesion and an adenocarcinoma. This set of experiments indicates that the NT-R in these tissues are of the type 1 subtype.

This study confirms on the one hand that pancreatic ductal cancers frequently express a high density of NT-R, as reported previously [1,2]. However, it expands this information in two ways: (1) it clearly shows that liver metastases also frequently express NT-R in high amounts. It thus closes an information gap and has clinical significance: indeed, the current knowledge of a NT-R expression solely in primaries would not be sufficient to legitimate NT-R targeted diagnostic or therapeutic interventions, since many of ductal pancreatic patients have metastases at the time of diagnosis. (2) The study also provides novel information on NT-R expression in putative pancreatic cancer precursor lesions, namely PanIN. It suggests an increase in NT-R expression from PanIN 1B to PanIN 3, with PanIN 1B completely lacking NT-R, as it is the case for normal pancreatic ducts [1], while PanIN 3 frequently expresses NT-R in high amounts. It is further of interest that in cases with NT-R-expressing PanIN 3, accompanying primary carcinomas also express NT-R, in line with the concomitant expression of many other biologic markers in high-grade PanIN and invasive cancer. Therefore, NT-R, in particular the type 1 NT-R detected in this study, may be considered as a novel, specific marker of pancreatic intraepithelial neoplasia of higher degree.

These data may have potential clinical implications. Indeed, knowing that NT stimulates pancreatic tumor growth [11,13,14] and that the type 1 NT-R-selective antagonist SR48692 inhibits tumor cell proliferation [13], it could be speculated that type 1 NT-R antagonists may be used for treatment of pancreatic cancer primaries, but also of their liver metastases and, speculatively, of the putative cancer precursors, the NT-R-expressing PanIN 2 and 3.

The data may also have more immediate diagnostic and radiotherapeutic implications. There are several newly designed neurotensin analogs which can be labeled with radioactive compounds [18,20,21,23] and that can be used either for SPECT or for PET diagnostic evaluation. Preliminary clinical evaluation was performed with two of them, however, with limited success probably due to rapid degradation of the tracers [27,28]. Recently, in line with the observation that somatostatin and bombesin receptor antagonists may be advantageous for tumor radiotargeting [31-34], radiolabeled NT-R antagonists have been developed aimed at greater metabolic stability, less side effects than agonists, and increased tumor uptake compared with agonists [25,26,29]. Such radioactive neurotensin analogs may become useful as diagnostics for the visualization of primary pancreatic carcinoma and their metastases and therefore help to decide on the therapeutic strategy. These radiolabeled neurotensin analogs may also be used for targeted radiotherapy of primaries and metastases, in particular since the NT-R expression is often very high. Moreover, at the same time, the putative precursor lesions, the NT-R-expressing PanIN 2 and PanIN 3, may also be destroyed. The amount of NT-R expressed in all these lesions appears clearly high enough for such a clinical application.

## Conclusions

These *in vitro* data extend the currently available information on NT-R in invasive and non-invasive pancreatic ductal tumors. They suggest that type 1 NT-R may be a novel, specific marker of PanIN of higher degree. The high expression of NT-R in primaries and metastases of invasive cancer strongly support the need to develop radioactive neurotensin analogs for the diagnosis and therapy of this tumor type.
